# DNA methylation of *SMPD3*-based diagnostic biomarkers of NASH and mild fibrosis

**DOI:** 10.1016/j.gendis.2023.03.023

**Published:** 2023-04-26

**Authors:** Na Wu, Mofan Feng, Siran Yue, Xinyu Shi, Nan Tang, Yalan Xiong, Jianying Wang, Lei Zhang, Hualing Song, Yi Shi, Guang He, Guang Ji, Baocheng Liu

**Affiliations:** a Shanghai Innovation Center of Traditional Chinese Medicine Health Service, School of Public Health, Shanghai University of Traditional Chinese Medicine, Shanghai 201203, China; b Institute of Digestive Diseases, Longhua Hospital, Shanghai University of Traditional Chinese Medicine, Shanghai 200032, China; c Bio-X Institutes, Key Laboratory for the Genetics of Developmental and Neuropsychiatric Disorders, Shanghai Jiao Tong University, Shanghai 200030, China; d Institute for Advancing Translational Medicine in Bone and Joint Diseases, School of Chinese Medicine, Hong Kong Baptist University, Hong Kong SAR 999077, China

Non-alcoholic fatty liver disease (NAFLD) is a liver condition that is widely prevalent across the world. A considerable number of people with NAFLD have the potential to progress to a more severe form of the condition known as nonalcoholic steatohepatitis (NASH), accompanied by bridging fibrosis. This advancement is more likely if the patient has metabolic risk factors such as obesity or type 2 diabetes that deteriorate over time. Additionally, even slight inflammation or fibrosis in NAFLD can significantly increase the likelihood of progression compared to steatosis alone. This underscores the importance of revising the present methods of monitoring NAFLD patients to ensure early detection and effective management of the disease. The pattern of DNA methylation varies with the onset of a wide range of NAFLD since it is extremely susceptible to internal and external environmental stressors; hepatic DNA methyltransferase has been connected to the NAFLD activity score (NAS) and has been demonstrated to have higher activity in NASH than simple steatosis.^1^ Thus, the current work aimed to clarify the role of DNA methylated genes, *i.e.*, sphingomyelin phosphodiesterase 3, *Smpd3* in liver tissue and adipose tissue of NASH and mild fibrosis, for identifying biomarkers for differentiating different stages of NAFLD and providing precision medicine approaches to NAFLD diagnosis and management.

Data on DNA methylation (GSE49542 and GSE48325, Illumina Infinium 450 k Human Methylation Beadchip) were collected and received from the GEO database at NCBI for this work. We first analyzed the DNA methylation data with the ChAMP data package. High-throughput chromosomal conformation capture (Hi-C) technology was used to create 3D genomic information from human embryonic stem cells.^2^ K nearest neighbors (KNN) was initially utilized to group differentially methylated genes from the perspective of gene structure/location according to the 3D genome of human cells since genes with neighboring chromosome positions have comparable functions (Fig. 1A; Fig. S1). After the Venn analysis, we found that NASH and mild fibrosis were both shown to have 36 overlapping differentially methylated genes (DMGs) (Fig. 1A, B and Table S1, 2). The above DMGs were given 3D genome coordinates based on the chromosomal number and TSS using our 3D modeling methods.^2^ These DMGs with 3D genomic information were then divided into four groups using KNN (optimal cluster numbers were pre-investigated). KEGG enrichment analysis was performed to investigate the biological role of the DMGs in NAFLD, and pathways with the assigned clusters based on 3D genome coordinates are provided in Table S3. We found that *SMPD3* and cytochrome P450 family 1 subfamily A member 1 (*CYP1A1*) were linked to the same cluster and implicated in the pathways for lipid metabolism (cluster 1). Despite possible batch effects between GSE48325 and GES49542, it is noteworthy that *SMPD3* (cg07735969) was hypermethylated in mild fibrosis compared to NASH (Table S4). An animal experiment verified the difference between *Smpd3* DNA methylation in NASH and mild fibrosis.

**Figure 1:**
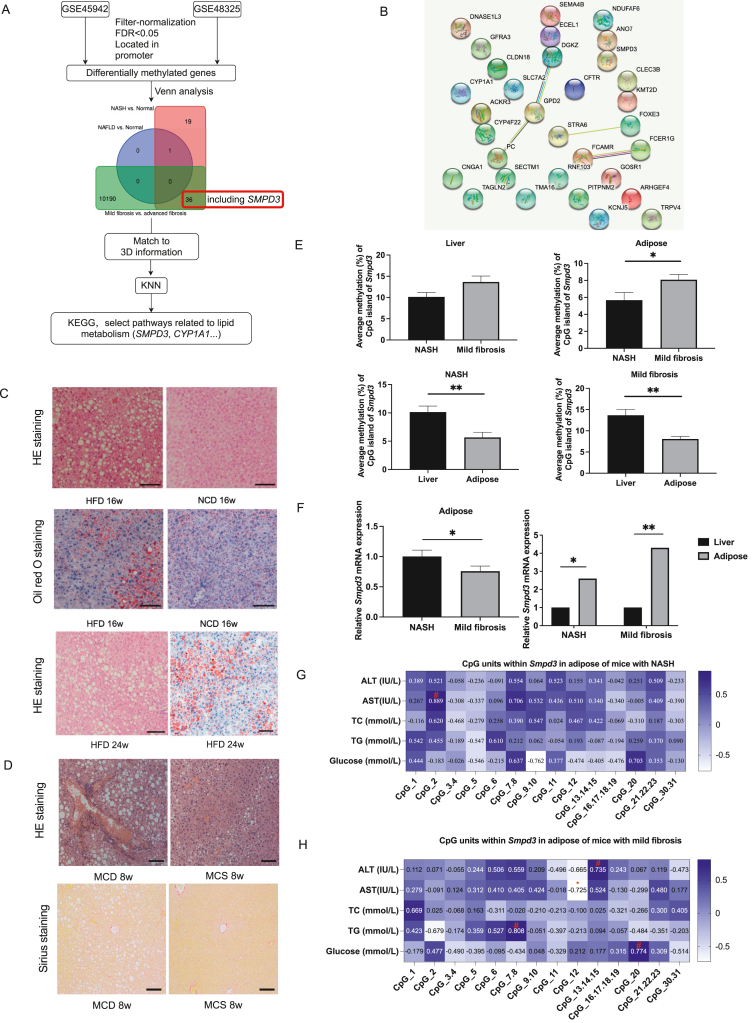
DNA methylation of *Smpd3* differs between NASH and mild fibrosis. **(A)** Flow chart of screening for DNA methylation analysis in NAFLD patients. **(B)** Network of overlapped differentially methylated genes in GSE48325 and GSE49542. **(C)** Histological assessment of the liver between NAFLD and NASH. Scale bars = 100 μm. **(D)** Histological assessment of the liver between NASH and mild fibrosis. Scale bars = 100 μm. **(E)** Average methylation (%) of *Smpd3* in the liver and adipose tissues of NASH and mild fibrosis mice. **(F)** Relative mRNA expression level of *Smpd3* in adipose tissue of NASH and mild fibrosis mice. ^∗^*P* < 0.05, ^∗∗^*P* < 0.01. **(G)** Correlation between clinical traits and methylation of CpG units within *Smpd3* in adipose tissue of NASH mice. **(H)** Correlation between clinical traits and methylation of CpG units within *Smpd3* in adipose tissue of mild fibrosis mice. # and ∗ indicate the significant positive and negative correlation, respectively (*P* < 0.05).

A high-fat diet (HFD) and a methionine choline-deficient diet (MCD), respectively, were used to create the NASH model and the mild fibrosis model in C57BL/6 male mice (specified pathogen-free (SPF) grade, aged 9 weeks) (Fig. 1C, D and Table S5, 6). The resultant DNA fragments were detected using matrix-assisted laser desorption/ionization time-of-flight mass spectrometry, and EpiTYPER (Agena Bioscience, San Diego, CA, USA) was employed to quantify the CpG methylation level of *Smpd3* in liver and adipose of NASH and mild fibrosis mice. NASH mice had significantly lower levels of CpG_1 methylation in their livers than mice with mild fibrosis (Table S7). The average methylation and methylation levels of CpG_1 and CpG_2 within *Smpd3* in adipose tissue were significantly decreased in NASH mice compared to mice with mild fibrosis (Fig. 1E, F and Table S7). We subsequently analyzed the DNA methylation profile between the liver and adipose tissue to determine whether *Smpd3* displayed tissue-specific DNA methylation. NASH mice had significantly greater levels of *Smpd3* mRNA expression in their adipose tissue than did mice with mild fibrosis (Fig. 1F). Additionally, average methylation of *Smpd3* was significantly higher in the liver than in adipose tissue in both NASH and mild fibrosis mice, and mRNA expression of *Smpd3* was significantly lower in the liver than in adipose tissue (Fig. 1E, F). Strong associations existed between methylation of CpG units within *Smpd3* and traits in adipose and liver of NASH and mild fibrosis explored (Fig. 1G, H; Fig. S2).

As one of the tumor suppressors linked to preventing cell proliferation and promoting tumors, *SMPD3*, also known as *nSMase2*, works as an enzyme to convert sphingomyelin into ceramide and phosphorylcholine. According to high throughput screening, human renal cell carcinoma had higher levels of *SMPD3* DNA methylation than the paired normal tissues.^3^ However, the relationship between NASH's methylation status and fibrosis has not yet been documented. In this study, we first investigated if DNA methylation of *SMPD3* from GEO might be a biomarker in NAFLD. Then we validated it using mouse data, consequently implying that methylation of *Smpd3* may distinguish between NASH and mild fibrosis. Given that *Smpd3* DNA methylation controls transcription activity in both liver and adipose tissues during the pathogenesis of NAFLD, the excellent concordance between GEO analysis and our mouse data further suggests that the DNA methylation profile of *Smpd3* seen in NASH and mild fibrosis is robust.

The activation or inhibition of gene expression induced by changes in DNA methylation of promoter regions is well established to have a significant role in NAFLD-related disorders. This study found that mild fibrosis-related adipose tissue had dramatically increased DNA methylation and decreased *Smpd3* expression. The survey by Kolak et al^4^ found that the *Smpd3* gene was expressed more in adipose tissue that was inflamed compared to adipose tissue that was not as inflamed. This suggests that the level of *Smpd3* expression is higher in tissues that are undergoing inflammation. This type of information is valuable as changes in gene expression of *Smpd3* can shed light on the differences between healthy and diseased states in a tissue. Additionally, the production of pro-inflammatory cytokines in adipocytes and macrophages can be increased through ceramides. Sphingomyelinase activity in adipose tissue has been shown to trigger inflammatory responses and activate macrophages. Thus, there is a strong correlation between sphingomyelinases and the accumulation of inflammation. This implies that there is an interdependent connection between *SMPD3* activity and inflammation. Notably, there is proof that NASH patients with fibrosis (stages 1, 2, 3, and 4) had a relative all-cause death rate of 1.6, 2.5, 3.5, and 6.4 times higher than those without fibrosis.^5^ It was recommended that NASH must be distinguished from mild or advanced fibrosis. As lobular inflammation and ballooning are the main factors promoting the development of liver fibrosis in NAFLD people, DNA methylation of *Smpd3* in the current study will be significant in risk stratification as a novel potential biomarker.

The study investigated how DNA methylation affected the expression of the *Smpd3* gene in NASH and mild fibrosis. It also emphasized how the DNA methylation of the *Smpd3* gene in NAFLD may serve as a biomarker to imitate an adipose tissue's epigenetic signature. All these findings offer comprehensive biological data for DNA methylation, which may aid in improving our comprehension of how NAFLD develops and progresses as well as in stratifying NASH into mild fibrosis and supplying methods for mild fibrosis treatment.

## Ethics declaration

The study was approved by the Experimental Animal Ethics Committee at the Shanghai University of Traditional Chinese Medicine.

## Author contributions

BL, GJ, and GH designed research; NW, MF, SY, NT, YX, XS, LZ, and JW conducted research; NW analyzed and interpreted data; NW wrote the manuscript; BL, GJ, GH, YS, and HS reviewed the manuscript critically. None of the authors reported a conﬂict of interest related to the study. All authors have read and agreed to the published version of the manuscript.

## Conflict of interests

All authors declare that there is no potential conflict of interests.

## Funding

This work was supported by a Three-year action plan for Shanghai (ZY(2021–2023)-0211), National Natural Science Foundation of China (No. 81973730), Local Colleges Faculty Constitution of Shanghai MSTC 2022 (No. 22010504300), Shanghai Collaborative Innovation Center for Chronic Disease Prevention and Health Services (2021 Science and Technology 02–37), China Postdoctoral Science Foundation, No. 72 General Fund, 2022 (No. 2022M722164), Shanghai 2023 "Science and Technology Innovation Action Plan" Qi Ming Xing Cultivation (Yang Fan Project, No. 23YF1447700), and Shanghai Health Commission for Traditional Chinese Medicine Research (No. 2022QN014).
