# Supplementary material for: DNA methylation of SMPD3-based diagnostic biomarkers of NASH and mild fibrosis
Source: Genes Dis. 2023 Apr 26;11(1):99–102. doi: 10.1016/j.gendis.2023.03.023 (PMC10425839; doi:10.1016/j.gendis.2023.03.023)
Supplement: Multimedia component 1 [file mmc1.docx]

**Supplementary Materials**

**Table S1.** Differentially methylated CpGs in GSE48325 and GSE49542

**Table S2.** Overlapping differentially methylated genes among normal, NAFLD, NASH, and fibrosis

**Table S3.** Pathways with the assigned clusters based on 3D genome coordinates

**Table S4.** Specific information on CpG sites of *SMPD3* in GSE49542 and GSE48325

|  | cg | Disease | Chromosome | Feature | CGI |
| --- | --- | --- | --- | --- | --- |
| GSE48325 |  | NASH |  |  |  |
|  | cg07735969 | 0.567 | 16 | 5’UTR | Open sea |
| GSE49542 |  | Mild fibrosis |  |  |  |
|  | cg07735969 | 0.765 | 16 | 5’UTR | Open sea |

CGI, cytosine-guanine dinucleotide island; CpG, cytosine-guanine dinucleotides; NASH, nonalcoholic steatohepatitis; Smpd3, sphingomyelin phosphodiesterase 3; 5’UTR, 5 prime untranslated regions.

**Table S5.** NAFLD activity score in the various groups based on HE staining

| Item | NAFLD (HFD 16w) | NASH (HFD 24w) | Mild fibrosis (MCD 8w) |
| --- | --- | --- | --- |
|  | Mean ± SD | Mean ± SD | Mean ± SD |
| Steatosis | 2.00 ± 0.76 | 2.63 ± 0.52 | 2.63 ± 0.76 |
| Lobular inflammation | 0.88 ± 0.35 | 2.25 ± 0.46 | 3.58 ± 0.35 |
| Ballooning | 1.02 ± 0.35 | 1.86 ± 0.38 | 1.88 ± 0.35 |
|  |  | ＞5 | ＞7 |

HE, haematoxylin and eosin; NAFLD, nonalcoholic fatty liver disease.

**Table S6.** Basic traits of NASH and mild fibrosis mice

|  | NASH | Mild fibrosis | p |
| --- | --- | --- | --- |
|  | Mean±SD | Mean±SD |  |
| Weight (g) | 51.11±6.13 | 15.18±0.75 | <0.001 |
| ALT (IU/L) | 224.63±100.08 | 101.63±30.08 | 0.005 |
| AST(IU/L) | 236.00±75.88 | 144.38±56.03 | 0.016 |
| TC (mmol/L) | 6.36±0.67 | 0.05±0.03 | <0.001 |
| TG (mmol/L) | 0.44±0.12 | 0.04±0.01 | <0.001 |
| Glucose (mmol/L) | 9.07±3.43 | 6.31±1.04 | 0.051 |

ALT, alanine aminotransferase; AST, aspartate aminotransferase; NASH, nonalcoholic steatohepatitis; TC, total cholesterol; TG, triglyceride.

**Table S7.** *Smpd3* methylation values (%) for each CpG unit among four groups in liver and adipose tissues of mice

| CpG units | Liver (Methylation%, Mean ± SD) | | p | Adipose (Methylation%, Mean ± SD) | | | p |
| --- | --- | --- | --- | --- | --- | --- | --- |
|  | NASH | Mild fibrosis |  | NASH | Mild fibrosis |  | |
| CpG_1 | 1.71 ± 0.64 | 7.88 ± 1.83 | 0.010 | 1.81 ± 0.50 | 4.88 ± 0.97 | 0.016 | |
| CpG_2 | 7.29 ± 2.71 | 12.00 ± 2.61 | 0.233 | 3.75 ± 2.26 | 3.13 ± 1.34 | 0.780 | |
| CpG_3.4 | 9.57 ± 5.02 | 11.75 ± 3.85 | 0.733 | 2.19 ± 0.76 | 7.88 ± 2.16 | 0.028 | |
| CpG_5 | 6.14 ± 3.04 | 5.50 ± 2.18 | 0.864 | 2.00 ± 1.05 | 8.75 ± 3.08 | 0.057 | |
| CpG_6 | 45.5 ± 17.73 | 49.33 ± 17.73 | 0.887 | 8.25 ± 4.22 | 13.5 ± 8.96 | 0.574 | |
| CpG_7.8 | 7.00 ± 3.59 | 8.14 ± 3.24 | 0.817 | 4.19 ± 1.66 | 3.38 ± 1.66 | 0.714 | |
| CpG_9.10 | 7.86 ± 2.65 | 10.75 ± 1.96 | 0.389 | 8.00 ± 1.78 | 10.88 ± 3.13 | 0.456 | |
| CpG_11 | 17.17 ± 4.66 | 12.43 ± 4.61 | 0.487 | 5.94 ± 1.90 | 11.38 ± 2.03 | 0.075 | |
| CpG_12 | 10.00 ± 3.93 | 9.75 ± 4.59 | 0.968 | 7.44 ± 2.22 | 6.75 ± 2.18 | 0.812 | |
| CpG_13.14.15 | 10.29 ± 2.14 | 8.37 ± 1.10 | 0.423 | 4.00 ± 1.29 | 6.50 ± 1.52 | 0.256 | |
| CpG_16.17.18.19 | 10.00 ± 1.60 | 16.50 ± 3.15 | 0.102 | 9.13 ± 1.96 | 13.12 ± 3.45 | 0.347 | |
| CpG_20 | 9.86 ± 2.47 | 19.88 ± 4.31 | 0.075 | 7.50 ± 2.04 | 4.50 ± 2.36 | 0.353 | |
| CpG_21.22.23 | 10.29 ± 2.90 | 15.00 ± 3.32 | 0.311 | 16.25 ± 3.20 | 13.75 ± 2.75 | 0.563 | |
| CpG_30.31 | 7.00 ± 0.90 | 11.00 ± 2.20 | 0.095 | 3.00 ± 0.90 | 6.00 ± 2.10 | 0.224 | |

NASH, nonalcoholic steatohepatitis.

**Figure S1.** Analyzed CpG sites methylation of promoter and CpG island

NASH, nonalcoholic steatohepatitis; TSS200, transcription start site 200; TSS1500, transcription start site 1500; 5’UTR, 5 prime untranslated region.

**Figure S2.** Correlation between clinical traits and methylation of CpG units within *Smpd3* in the liver of NASH and mild fibrosis mice

ALT, alanine aminotransferase; AST, aspartate aminotransferase; NASH, nonalcoholic steatohepatitis; Smpd3, sphingomyelin phosphodiesterase 3; TC, total cholesterol; TG, triglyceride. # and * indicate the significant positive and negative correlation, respectively (p<0.05).

**Materials and methods**

***Microarray data***

Data on DNA methylation (GSE49542 [1] and GSE48325 [2], Illumina Infinium 450k Human Methylation Beadchip) were collected and received for this work from the GEO database at the National Center for Biotechnology Information (Gene Expression Omnibus, https://www.ncbi.nlm.nih.gov/geo/). Fifty-six liver biopsies from patients with biopsy-proven NAFLD were included in GSE49542, including 33 with mild and 23 with advanced fibrosis. In GSE48325, there were 18 samples of healthy controls, 14 samples of NASH, and 12 samples of steatosis.

***Data process***

*Genome-wide DNA methylation analysis*

R and Bioconductor programs were used to pre-process the raw DNA methylation data. The ChAMP data package was used to process the output Idat files (version 2.8.1). Several filtering procedures were carried out after loading the Idat files: 1). Samples with probe ratios greater than the default (0.1) threshold should be discarded; 2) Remove all non-CpG probes from this dataset and any probes with fewer than three beads that are present in at least 5% of the samples for each probe; 3) To increase hybridization efficiency and discover the real signals, exclude all SNP-related probes, multi-hit probes, or probes found on chromosomes X and Y [3, 4]. Next, the effects of type-II probe bias were adjusted using the BMIQ function. We used the false discovery rate (FDR) on our genome-wide analysis of DNA methylation (q < 0.05) to account for multiple testing and reduce the number of false positives, and CpG sites that are located in promoters (transcription start site 200 (TSS200), TSS1500, and 5 prime untranslated region (5’UTR)) were used for further analysis (|Δβ| ≥ 0.1), as there is undisputed evidence that methylation at promoters silenced [5]. Genes with the relevant CpGs in their promoters were identified as differentially methylated genes (DMGs). Venn analysis was used to examine the overlapping DMGs in the various stages of NAFLD (NASH and mild fibrosis) from the screening from GES49542 and GSE48325 (Figure 1A).

*3D genome [6], functional and pathway enrichment analysis*

High-throughput chromosomal conformation capture (Hi-C) technology was used to create 3D genomic information from human embryonic stem cells [7]. K nearest neighbors (KNN) was originally utilized to group differentially methylated genes from the perspective of gene structure/location according to the 3D genome of human cells since genes with neighboring chromosome positions have comparable functions [8]. Then, using the R program clusterProfiler [9], KEGG pathway enrichment analysis was carried out, and a q value of less than 0.05 was regarded as statistically significant. The most DMGs may be chosen by using this two-pronged technique along with 3D genome data and KEGG enrichment analysis (Figure 1A).

**Animal experiment**

***Modeling stage***

A high-fat diet (HFD) and a methionine choline-deficient diet (MCD), respectively, were used to create the NASH model and the mild fibrosis model in C57BL/6 male mice with specified pathogen-free (SPF) grades, aged 9 weeks. After acclimating to their surroundings for a week, all animals had free access to food and tap water at a pathogen-free animal care facility, provided their weight was under 25 g. The mice were randomized to either an HFD (#D12492, Research Diet, n = 11) or a normal chow diet (NCD, n = 11) for 24 weeks at 10 weeks of age. To determine whether the mice were NAFLD animals, 3 mice from each group were slaughtered after 16 weeks of HFD; 2) for 8 weeks, either a methionine choline sufficient diet (MCS, #519581, Dyets, n = 8) or an MCD (#519580, Dyets, n = 8). After that, the mice were sacrificed, and the blood was taken. The serum was then refrigerated at -80°C for the biochemical analysis. Weights were taken off the epididymal and liver fat tissues. Snap-frozen aliquots were kept at -80°C in cold storage.

***Histological examination***

A portion of the liver tissue was processed, embedded in paraffin, and preserved in 4% buffered paraformaldehyde for histological examination. Haematoxylin and eosin (HE), oil red O, or Sirius red were used to stain liver slices for routine histology, lipid detection, or collagen deposition.

A neutral glue was used to seal off liver sections, which were then examined and captured on camera under a microscope. The liver slides were then examined with the NAS for steatosis, inflammation, and fibrosis. Steatosis (0-3 points), lobular inflammation (0-3 points), and hepatocyte ballooning (0-2 points) were each given individual scores, and the combined score was then calculated in a blinded manner. A NAS score of less than 3 is not considered a NASH condition, whereas a score of more than 5 indicates NASH [10].

***Biochemical assay***

Utilizing an automated clinical chemistry analyzer (7020, HITACHI, Japan) in the Laboratory of Animal Centre, Shanghai University of Traditional Chinese Medicine, the following parameters were determined: fasting glucose, total cholesterol (TC), triglyceride (TG), serum alanine transaminase (ALT), and aspartate transaminase (AST) levels.

***DNA methylation analysis***

A TIANamp Genomic DNA Kit (#DP304, TIANGEN, China) was used to isolate genomic DNA from mouse liver and adipose tissue. The EpiTect Fast Bisulfite Conversion Kit (#59824, Qiagen, Germany) was used to bisulfite converted genomic DNA. The region of the CpG island was chosen to examine the CpG methylation level. The EpiDesigner program was used to create target-specific primer pairs to amplify genomic DNA that had undergone bisulfite treatment (Agena Bioscience, Inc., San Diego, CA, USA). Each reverse primer featured a T7 promoter tag for transcription (5'-CAGTAATACGACTCACTATAGGGAAGGC T-3') and was created by General. A 10-mer (5'-AGGAAGAGAG-3') was added to the forward primer to balance the melting temperature. Shrimp alkaline phosphatase was used to treat the PCR-amplified products, and concurrent in vitro transcription and base-specific cleavage were carried out. The SMPD3 primers were as follows, yielding 280 bp fragments: forward: aggaagagagGGAATTTTTTAATAGGTAATTTTGG; reverse: cagtaatacgactcacta tagggagaaggctATCCTTAACACCCACCTCAAATAAC. The resultant DNA fragments were detected using matrix-assisted laser desorption/ionization time-of-flight mass spectrometry, and EpiTYPER (Agena Bioscience, San Diego, CA, USA) was employed to quantify the CpG methylation level.

***Quantitative real-time PCR analysis* (qRT-PCR)**

TRIzol reagent (#15596-026, Invitrogen, USA) was used to extract the total RNA from the liver and adipose tissue in accordance with the manufacturer's instructions. The Hifair® II 1st Strand cDNA Synthesis Kit (#HB200724, YEASON, China) was used to create cDNA from 2 g of total RNA. SYBR Premix Ex Taq (Tli RNaseH Plus) (#RR420A, TaKaBa, Japan) was used to amplify total cDNA. A LightCycler was used to conduct the PCRs (Roche Diagnostics, USA). Each sample was examined three times. Gene expression was expressed as 2(^-ΔΔCt^) and normalized to the housekeeping gene *β-Actin*, forward: CCCAGCACAATGAAGATCAAGATCAT; reverse: ATCTGCTGGAAGGTGGACAGCG. The sequences of the primers used were as follows: *SMPD3* (forward: GCTGAGGTGGTATAGGTCCTG; reverse: CACCGGAGTCTGCTGAAAAC).

***Statistical analysis***

The mean, standard deviation (SD), or standard error of basic mouse characteristics were shown. For the comparison of the two groups, independent samples t-test was used. T-tests were performed to compare the two groups. For pairwise comparisons across groups, the least significant difference (LSD) test [11] was used with the presumption that the variance was uniform. Non-normally distributed data were evaluated by converting logs to normally distributed data. For data that could not be transformed into normally distributed data, non-parametric testing, such as the Mann-Whitney U and Kruskal-Wallis H test, was utilized. Spearman correlation analysis was used to determine the relationship between serum characteristics or mRNA expression and CpG unit methylation. Statistical significance was set at P < 0.05.

**Reference**

1. Murphy SK, Yang H, Moylan CA, et al. Relationship between methylome and transcriptome in patients with nonalcoholic fatty liver disease. Gastroenterology*.* 2013;145:1076-1087.
2. Ahrens M, Ammerpohl O, von Schonfels W, et al. DNA methylation analysis in nonalcoholic fatty liver disease suggests distinct disease-specific and remodeling signatures after bariatric surgery. Cell Metab*.* 2013;18:296-302.
3. Zhou W, Laird PW, Shen H. Comprehensive characterization, annotation and innovative use of Infinium DNA methylation BeadChip probes. Nucleic Acids Res*.* 2017;45:e22.
4. Chen YA, Lemire M, Choufani S, et al. Discovery of cross-reactive probes and polymorphic CpGs in the Illumina Infinium HumanMethylation450 microarray. Epigenetics*.* 2013;8:203-209.
5. Jones PA. Functions of DNA methylation: islands, start sites, gene bodies and beyond. Nat Rev Genet*.* 2012;13:484-492.
6. Dixon JR, Selvaraj S, Yue F, et al. Topological domains in mammalian genomes identified by analysis of chromatin interactions. Nature*.* 2012;485:376-380.
7. Shi Y, Guo Z, Su X, et al. DeepAntigen: a novel method for neoantigen prioritization via 3D genome and deep sparse learning. Bioinformatics*.* 2020;36:4894-4901.
8. Dekker J, Marti-Renom MA, Mirny LA. Exploring the three-dimensional organization of genomes: interpreting chromatin interaction data. Nat Rev Genet*.* 2013;14:390-403.
9. Yu G, Wang LG, Han Y, et al. clusterProfiler: an R package for comparing biological themes among gene clusters. OMICS*.* 2012;16:284-287.
10. Kleiner DE, Brunt EM, Van Natta M, et al. Design and validation of a histological scoring system for nonalcoholic fatty liver disease. Hepatology*.* 2005;41:1313-1321.
11. Klein G, Dabney A. *The cartoon introduction to statistics*: Hill and Wang, a Division of Farrar, Straus and Giroux; 2013.
